# Ethnic differences in right ventricular structure and function in urbanized hypertensive patients in the Gornaya Shoriya region

**DOI:** 10.1038/s41598-023-31834-y

**Published:** 2023-03-21

**Authors:** Alexey N. Sumin, Nina S. Gomozova, Anna V. Shcheglova, Oleg G. Arkhipov

**Affiliations:** 1 Laboratory of Comorbidity in Cardiovascular Diseases, Federal State Budgetary Institution “Research Institute for Complex Issues of Cardiovascular Diseases”, 6, Sosnoviy Blvd, Kemerovo, Russian Federation 650002; 2 Myski City Hospital, Polyclinic No. 1, 23, Pervomajskaya St., Myski, Kemerovo Region Russian Federation 652849; 3 Ultrasound doctor, Individual entrepreneur, 5, Mira St., Myski, Kemerovo region Russian Federation 652845

**Keywords:** Cardiology, Signs and symptoms

## Abstract

Aim of this study was to compare right ventricular echocardiography parameters in urbanized hypertensive patients of the Shor and non-indigenous ethnic groups in the Mountain Shoria region. The study included patients with arterial hypertension: 58 Shors and 50 non-indigenous urbanized residents, comparable in age, and divided by ethnicity and gender into 4 groups: Shors men (n = 20), Shors women (n = 38) , non-indigenous men (n = 15) and non-indigenous women (n = 35). All underwent echocardiographic examination, and the right heart parameters were studied. Shor men with arterial hypertension had the lowest values ​​of the pulmonary artery index (*p* = 0.05), the right atrium dimensions (*p* = 0.04), and the highest values ​​of the blood flow velocity in the right ventricle, et' (*p* = 0.05) and st' (*p* = 0.05) in comparison with non-indigenous men. Shor women have the lowest values Et/At ratios (*p* = 0.05). RV diastolic dysfunction was detected mainly in women compared with men (23.1% and 1.9%, *p* = 0.0014), somewhat more often in Shors. Ethnicity was one of the factors associated with the right ventricular diastolic dysfunction presence (*p* = 0.002). Among the factors associated with the RV diastolic dysfunction were risk factors (smoking, obesity), blood pressure, gender, ethnicity, and left ventricular parameters (diastolic dysfunction and the myocardial mass increase). Thus, our study established the influence of ethnic differences on the right heart echocardiographic parameters in Shors and Caucasians with arterial hypertension. The effect of sex on RV diastolic dysfunction was a lot bigger compared to the effect of ethnicity. The revealed differences should improve the assessment of the right heart structure and function in patients with arterial hypertension from small ethnic groups, which will help to improve the diagnosis and treatment of such patients.

## Introduction

Recent studies have pointed out an unfavorable course of arterial hypertension in European ethnic minority populations compared to host populations^1,2^. A similar situation is observed when examining ethnic minority populations in other regions, for example, in Russian Siberia^3,4^. To improve treatment and control rates and reduce differences between populations, it is necessary to identify the determinants of these rates across ethnic groups, and to develop and implement ethnicity-specific intervention strategies, which ultimately help reduce ethnic disparities in hypertension-related complications^2^.

There are more and more data on importance of the right heart’s clinical and prognostic significance in cardiac diseases^5–7^. However, this information mainly relates to chronic heart failure, coronary artery disease or valvular diseases^5,6,8–10^. Assessment of right ventricular (RV) function is still insufficiently studied in patients with arterial hypertension, despite the first information about its dysfunction in hypertension being obtained over 30 years ago^11^. Researchers are currently studying the factors associated with RV remodeling in hypertension^12^ and have already shown an independent effect of RV parameters on the prognosis in these patients^13^. Distinguishing between normal and abnormal right heart dimensions and functions is therefore clinically relevant. However, a routine assessment of RV function in hypertensive patients (as opposed to, for example, assessing the state of the left ventricle (LV)) has not yet been widely used in clinical practice. Limited normative data is one of the possible barriers: developing normative indicators for right heart echocardiographic assessment used the results of studies carried out mainly in Caucasians in Europe and North America^14,15^, which cannot be extended to other racial and ethnic groups. Several recent multicenter studies have been aimed primarily at obtaining information about the ethnic characteristics of the left heart structural and functional indicators^16–18^. For the right heart, such studies are extremely scant^19,20^, therefore, the study of right heart ethnic characteristics in normal and pathological conditions remains an important scientific direction.

However, there is another aspect of the problem. Changes in the structure and function of the heart may not only be caused by genetic characteristics of a particular ethnic group, but also by peculiarities of the lifestyle (for example, traditional lifestyle of ethnic groups), living conditions (countryside / city, highlands / plain), as well as influence of pathological conditions. Previous studies have studied the features of echocardiography in representatives of two ethnic groups of the Gornaya Shoria region—the indigenous population (Shors) and the non-indigenous population (Caucasians) living both in rural areas^3,4^ and in the city^21^, (Sumin et al., in press). While the ethnic differences in left ventricular echocardiographic parameters were studied both for healthy urban residents of these groups^21^ and for patients with arterial hypertension (Sumin AN, in press), the indicators of the right ventricle were only compared in healthy individuals^21^. Accordingly, the aim of this study was to compare right ventricular echocardiography parameters in urbanized hypertensive patients of the Shor and non-indigenous ethnic groups.

## Methods

### Patients

**Figure 1:**
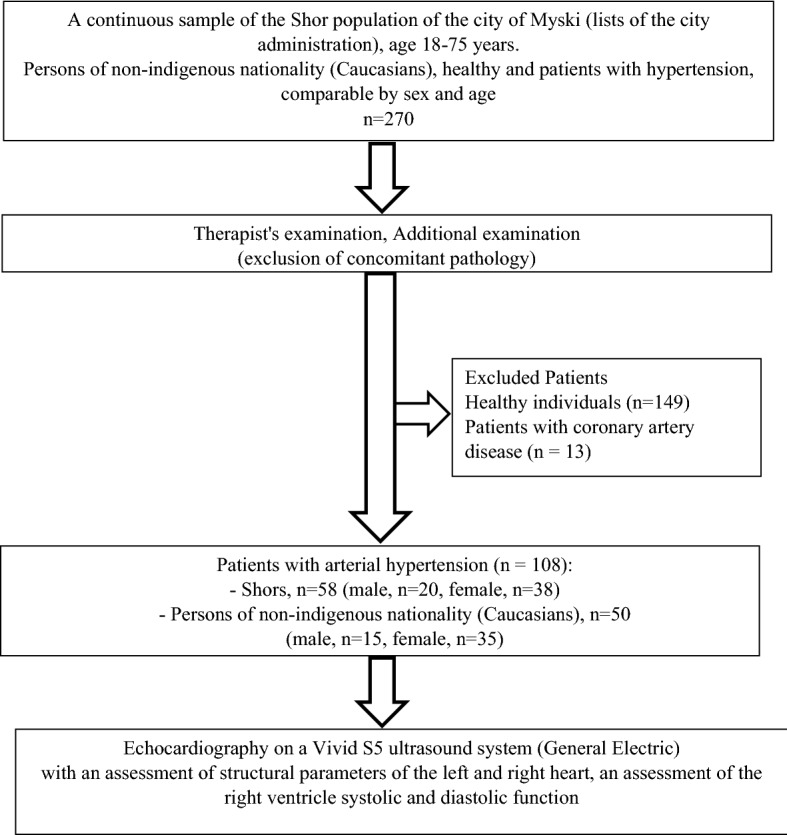
Study flowchart.

A cross sectional study of the indigenous (Shor) and non-indigenous (Caucasians) population living in Gornaya Shoria in the south of Western Siberia was carried out during 2017 and 2018. The Shors belong to the South Siberian segment of the Asian race^3^; in small rural communities in the middle mountains, their lifestyle is focused on hunting, fishing, subsidiary animal husbandry, primitive manual farming and gathering. Following intensive urbanization, the Shors are resettling from the countryside to the cities, which changes the usual way of life. The present study included Shors living in urban conditions. Recruitment of the studied indigenous nationalities and comparison groups was carried out by a continuous method according to the lists provided by the Myski city administration (Fig. 1), with persons aged 18–55 years. Initially, 270 adults (154 Shors and 116 non-indigenous residents) were examined by a therapist during a visit to the city polyclinic. Office blood pressure was measured using an Omron X3 Comfort (HEM-7155-EO) (OMRON, Kyoto, Japan); arterial hypertension was defined as a systolic blood pressure of 140 mm Hg or more, diastolic blood pressure 90 mm Hg or more and / or taking antihypertensive drugs. Patients with symptoms of angina pectoris were excluded from the study; a total of 108 patients with arterial hypertension (58 Shors and 50 Caucasians subjects) were identified. Ethnic groups were matched for gender and age. The study was carried out in accordance with the Helsinki Declaration, approved by the Local Ethics Committee of the Research Institute for Complex Issues of Cardiovascular Diseases (Kemerovo, Russian Federation) and all study participants signed an informed consent.

### Echocardiography

All subjects underwent echocardiography on a Vivid S5 ultrasound system (GE Healthcare, Chicago, Illinois, USA) using a phased array sector probe. All echocardiographic examinations were performed by the same examiner (OA), measurements were carried out according to the current guidelines^14,15^. Reported values of all echocardiographic parameters were obtained as the average value of 3 consecutive cardiac cycles. Structural parameters of the left and right heart were assessed using M-modal and B-modal scanning in standard positions. All structural indicators were indexed to the BSA. The following left heart indicators were used: end-diastolic and end-sistolic diameters (EDD and ESD), posterior wall thickness (TPWLV) and its index (TPWLVi), interventricular septum thickness (TIVS) and its index (TIVSi), end-diastolic and end-systolic volumes (EDV and ESV) of the LV, index of the end-diastolic volume of the LV (LVEDVi). LV mass was calculated in B-mode at the end of diastole, and LV myocardial mass index (LVMi) was also calculated. The LV ejection fraction (LVEF) was determined by the Simpson method. In the section of 4 chambers at the end of diastole, the maximum transverse diameter of the left atrium (LA), its volume (LAV) and index (LAVi) were measured. In the pulse-wave Doppler mode, the velocity of early diastolic atrioventricular flows (E), the flow rate of atrial systole (A). Using spectral tissue Doppler, the parameters of the regional function of the mitral valve annulus, related to the diastolic (e ', a') and systolic parts of the spectrum (s'), were measured.

From the data of the right heart, the diameter of the pulmonary artery (PA), its index (PAi), the end-diastolic anteroposterior size of the right ventricle (RV), its index (RVi) were assessed; the size of the right atrium (RA), its area (RAS) and the area index (RASi). In the M-mode, the longitudinal systolic function of the RV was assessed by measuring the systolic excursion of the tricuspid annulus (TAPSE).

In the pulse-wave Doppler mode, the velocities of early diastolic atrioventricular flows (Et), the time of their deceleration (DTt), the flow rates of atrial systole (At), the ratio Et / At were assessed. We assessed the parameters of the regional function of the tricuspid valve annulus, related to the diastolic (e't, a't, e't / a't), and the systolic part of the spectrum (s't). The index of the overall performance of the RV were calculated as the ratio of the sums of the isovolumic relaxation time and the isometric filling time to the expulsion time (Tei index). Using color M-modal scanning, we measured the propagation velocities of the early tricuspidal flow (Vft) according to the slope of the brightest part of the spectrum. RV diastolic dysfunction was considered with the ratio Et/At < 0.8 or > 2.1 and/or the ratio Et/e’t > 6^14^.

Since the assessment of the RV has not been well established, we used a sufficient number of right heart measurements to detect possible ethnic differences. In assessing the systolic function of the right ventricle, we focused primarily on the indicators of s't and TAPSE. Right ventricular diastolic function was assessed using the ratio Et/At, the ratio Et/et’, Vft, and the ratio e’t/a’t based on our previous studies^7,10^.

### Statistical analysis

Statistical processing was performed using the standard Statistica 10.0 and SPSS 17.0 software packages. Qualitative values were presented in absolute numbers (n) and percentage (%), comparisons between the groups were performed using χ2 tests. The normality of the distribution was verified using the Kolmogorov–Smirnov test. For a distribution other than normal, all quantitative variables were presented as the median, low and upper quartiles (ME [LQ, UQ]). Comparison of quantitative data was carried out using the Kruskal—Wallis test. Qualitative and binary characteristics were compared using the χ2 (chi-square) test with Yates' correction for small samples. Intergroup differences were assessed using the Mann—Whitney test with Bonferroni's correction. Using binary logistic regression analysis (enter method), we studied the relationship of possible factors with RV diastolic dysfunction. The level of statistical significance was taken as *p* < 0.05. Performance of RV values for diagnosing the RV diastolic dysfunction presence was assessed through receiver operating characteristic curve analysis. For intra-observer variability, the analysis was repeated 1 week after the first measurement in 20 random patients. Reproducibility was expressed using the coefficient of variation and interclass correlation coefficients.

## Results

General characteristics of the Shor and Caucasian groups are presented in Table 1. The groups were comparable in terms of age and sex. Anthropometric indicators (height, weight, BSA) were lower in Shors compared to Caucasians, but the differences did not reach statistical significance including for BMI (*p* = 0.069). Smoking was more common among the Shors (*p* = 0.012). Obesity, and office diastolic blood pressure levels were higher among the non-indigenous population (*p* = 0.028, and *p* = 0.028, respectively). The Shors had significantly lower triglyceride, LDL and urea levels and higher HDL levels than the non-indigenous population. A more detailed description of these groups, depending not only on ethnicity, but also on gender, was presented by us earlier (Sumin et al., in press).

**Table 1:** General characteristics of hypertensive patients of various ethnic groups.

	Shors (n = 58)	Non-indigenous ethnicity (n = 50)	Z	p
Age (years)	49 [44–54]	52 [44–55]	− 0.974	0.330
Men, n (%)	20 (34.5%)	15 (30%)	− 0.401	0.689
Weight (kg)	68 [62–80]	78 [67–88]	0.155	0.877
Height (cm)	158.5 [ 155–164]	163 [158–167]	1.206	0.228
BSA (m^2^)	1.74 [1.67–1.85]	1.86 [1.72–1.99]	− 0.039	0.968
BMI (kg/m^2^)	27.67 [24.03–30.80]	30.41 [25.67–32.56]	− 1.818	0.069
Obesity, n (%)	18 (31%)	26 (52.0%)	− 2.201	0.028
Elementary education, n (%)	4 (6.9%)	1 (2%)	0.437	0.662
Secondary education, n (%)	23 (39.7%)	12 (24%)	1.399	0.162
Secondary special education, n (%)	28 (48.3%)	34 (68%)	− 1.762	0.078
University education, n (%)	3 (5.2%)	3 (6%)	− 0.074	0.941
Hard physical labor, n (%)	36 (62%)	23 (46%)	1.436	0.151
Smoking, n (%)	23 (39.7%)	9 (18%)	− 2.446	0.014
SBP (mm Hg)	138.4 ± 17.4	145.4 ± 16.9	− 1.879	0.060
DBP (mm Hg)	81.9 ± 7.6	86.6 ± 9.6	− 2.203	0.028
Glucose (mmol / L)	4,95 [4,22–5,80]	5.92 [5.15–6.20]	− 3.441	0.0006
Cholesterol (mmol / L)	6.47 [5.60–6.97]	6.40 [5.90–7.16]	− 0.78250	0.433924
LDL (mmol / l)	2.90 [2.16–3.70]	3.71 [2.90–4.15]	− 2.298	0.022
HDL (mmol / L)	1.17 [1.0–1.59]	1.06 [0.94–1.20]	− 3.364	0.0008
Triglycerides (mmol / L)	2.10 [1.49–2.70]	2.44 [2.10–2.94]	2.083	0.037
Urea (mmol / L)	4.82 [3.70–6.41]	6.80 [5.60–7.49]	− 4.402	0.00001
Creatinine ( μmol / l )	87.0 [75.1–113.0]	94.0 [82.1–109.0]	− 0.736	0.462
ACE inhibitors, n (%)	17(29.3)	33(66.0)	− 2,786	0.0011
β-blockers, n (%)	7(12.1)	14(28.0)	− 1,218	0.092
Diuretics, n (%)	2(3.44)	(4.0)	0,0039	0.98
Regular medication, n (%)	14 (24.14)	30 (60.0)	3.24	0.00015

SBP—systolic blood pressure; DBP—diastolic blood pressure, BSA—body surface area, BMI—body mass index; LDL-low-density lipoprotein; HDL—high density lipoprotein; ACE—angiotensin converting enzyme.

The main structural indicators of the left ventricle, stratified by sex, had no ethnic differences (Table 2). Only the E / A ratio and the Tei index were the highest in the Shor men (*p* = 0.016 and *p* = 0.034, respectively).

**Table 2:** Left ventricular and atrial parameters in hypertensive patients of various ethnic groups.

	Patients of Shor nationality (n = 58)		Patients with non-indigenous ethnicity (n = 50)		p
	Men (n = 20)	Women (n = 38)	Men (n = 15)	Women (n = 35)	
TPW (mm)	11 [11.0–12.0]	12 [11.0–13.0]	11 [11.0–12.0]	11 [11.0–13.0]	0.38
TPWi (mm/m^2^)	9.06 [8.5–10.3]	8.6 [7.2–10.3]	8.8 [6.6–9.7]	8.5 [7.5–9.6]	0.15
TIVS (mm)	12 [11.0–13.0]	12.5 [11.0–13.0]	11 [11.0–12.0]	12.0 [10.0–13.0]	0.18
TIVSi (mm/m^2^)	9.8 [7.9–10.7]	9.08 [8.09–10.4]	8.8 [6.6–9.7]	8.3 [7.2–9.5]	0.39
LVMi (g/m^2^)	141.9[119.7–164.2]	132 [114–162]	127.4 [105–147]	134 [108.4–154]	0.37
LVEDD (mm)	50 [48–53]	53 [48–54]	55.5 [53–56]	51 [49–55]	0.30
LVEDVi (ml/m^2^)	89.4 [84–98]	86.7 [73.5–110]	102 [77.9–120]	89.8 [79.5–104.6]	0.46
LVEF %	66 [62–69]	65 [63–68]	67 [64–70]	67 [64–70]	0.52
LA (mm)	38 [35–39]	38 [37–40]	39 [37–40]	39 [37–40]	0.26
LAV (ml)	81 [50–89]	79 [57–88]	89 [68–91]	81 [66–89]	0.68
LAVi (ml/m^2^)	63.2 [41.9–72.5]	54.5 [44.5–62.5]	64.3 [47.8–69.7]	53.1 [40.4–66.4]	0.14
E/A	1.3 [1.1–1.54]	0.87 [0.75–1.15]	0.99 [0.63–1.28]	0.81 [0.71–1.27]	0.016
s' (cm/s)	9.5 [9.0–12.0]	9.0 [7.0–10.7]	9.0 [7.8–13.0]	10.0 [8.0–12.0]	0.559
E/e'	0.37 [0.30–0.44]	0.30 [0.26–0.41]	0.37 [0.29–0.47]	0.32 [0.28–0.47]	0.44
LV Tei index	0.37 [0.29–0.44]	0.49 [0.40–0.58]*	0.49 [0.42–0.73]*	0.50 [0.39–0.64]	0.034

TPW—posterior wall thickness, TIVS—interventricular septum thickness LVM—LV myocardial mass, LVEDD—left ventricular end-diastolic diameter, LVEDV—left ventricular end-diastolic volume, LVEF—left ventricular ejection fraction LA—left atrium, LAV—left atrium; All structural indicators were indexed to the body surface area; E—early diastolic mitral flow (pulse Doppler); A—late diastolic mitral flow (pulse Doppler); e’ early diastolic relaxation velocity, s’—systolic velocity of lateral mitral annulus (tissue Doppler), LV—left ventricular; *p < 0.05 compared with Shor men.

Right ventricle parameters had more noticeable ethnic differences (Table 3). In Shor men, the lowest values of the pulmonary artery index (*p* = 0.05), right atrium sizes (*p* = 0.04), and the highest values of the propagation velocity of the RV filling flow (*p* = 0.01), velocity of early diastolic (*p* = 0.05) and systolic (*p* = 0.05) tricuspid annulus movement were identified. Shor women showed the smallest values of the Et / At ratio (*p* = 0.05).

**Table 3:** Right ventricular and atrial parameters in hypertensive patients of various ethnic groups.

	Patients of Shor nationality (n = 58)		Patients with non-indigenous ethnicity (n = 50)		H	p
	Men (n = 20)	Women (n = 38)	Men (n = 15)	Women (n = 35)		
PA (mm)	21 [20–21]	21 [20–22]	22 [20–22]	21 [20–22]	3.88	0.16
PAi (mm/m^2^)	17.2 [15–18]	15.7 [13.9–17.9]*	15.3 [12.6–17.6]	15.4 [13–17.4]	5.47	0.05
RA (mm)	32 [31.5–35]	35 [32–36]*	35 [33–38]*	35 [32–38]	7.59	0.04
RAS (cm^2^)	11.5 [11.5–14.4]	14.4 [11.5–16.3]	15.4 [12–17.5]	14.2 [11.5–17]	5.35	0.067
RASi (cm^2^/m^2^)	10 [8.4–11.7]	10.3 [8.7–11.7]	10.7 [9.3–12]	9.3 [7.6–11.5]	2.56	0.78
RV (mm)	29 [25–32]	30 [28–32]	30 [29–30]	30 [29–31]	1.33	0.51
RVi (mm/m^2^)	16.74 [14.09–18.19]	16.22 [15.14–18.18]	15.64 [14.41–17.11]	16.52 [15.34–17.53]	1.46	0.48
TAPSE (mm)	25 [21–26.5]	23 [19–27]	21 [19–25]	20 [18–25]	4.3	0.3
Et (cm/s)	55 [49–65.5]	47.5 [43–60]	53 [46–62]	53.5 [50–59]	− 1.84	0.06
At (cm/s)	43 [32.3–51.2]	46.5 [35.3–54.8]	38.7 [34.8–45.9]	42 [34.0–50.9]	0.64	0.52
Et/At	1.26 [1.1–1.6]	1.11 [1.0–1.34]	1.25 [1.09–1.45]	1.27 [1.1–1.47]	− 1.95	0.05
DT_t_ (ms)	187.5 [152.5–229.5]	196 [170–251]	178.5 [130–207]	180 [163–222]	0.88	0.37
V_ft_ (cm/s)	43.5 [39–47]	37 [34–44]*	39 [37–52]	38 [33–49]	− 2.43	0.01
RV IVRT (ms)	72 [67–78]	70 [67–80]	80 [72–85]	75.5 [67.5–81.5]	0.09	0.92
e'_t_ (cm/s)	16.0 [11.0–18.0]	13.0 [9.0–16.7]	13.7 [11.1–18.0]	12.8 [10.2–16.0]	− 1.93	0.05
a'_t_ (cm/s)	15.9 [13.4–21.6]	14.6 [11.3–18.03]	17.4 [12.9–19.2]	15.0 [12.2–17.5]	− 1.03	0.30
e'_t_ / a'_t_	1.04 [0.68–1.3]	0.8 [0.7–0.95]	0.8 [0.68–1.04]	0.8 [0.73–1.04]	− 1.81	0.07
s'_t_ (cm/s)	17.0 [13.8 − 19.5]	13.0 [11.0–17.9]	16.0 [11.0–20.0]	14.0 [11.0 − 15.7]	− 0.95	0.05
RV Tei index	0.48 [0.3–0.5]	0.39 [0.3–0.5]	0.5 [0.4–0.5]	0.47 [0.3–0.6]	0.37	0.94

PA—pulmonary artery diameter; RAS—right atrium square; RV—right ventricle; TAPSE—tricuspid annular plane systolic excursion; RA—right atrium; mPAP—mean pulmonary artery pressure; SPAP—systolic pulmonary arterial pressure; E_t_—early transtricuspid diastolic filling; A_t_—late transtricuspid mitral diastolic filling; e'_t_—early diastolic tricuspid annular tissue velocity; a'_t_—late diastolic tricuspid annular tissue velocity; s'_t_—systolic tricuspid annular tissue velocity; *p < 0.05 compared with Shor men.

**Figure 2:**
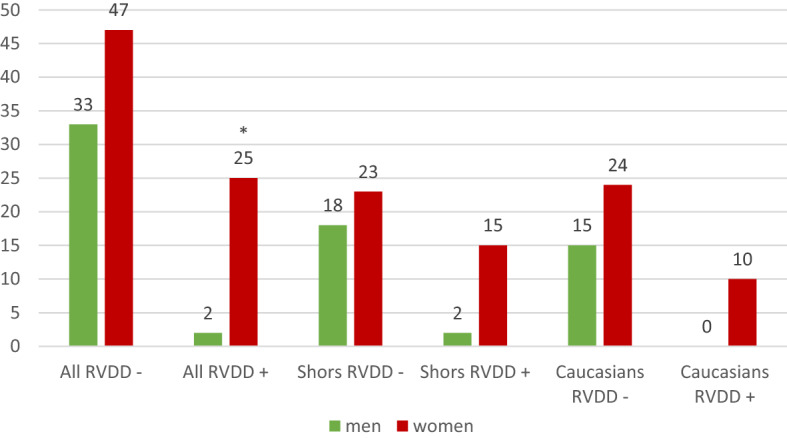
The right ventricle diastolic dysfunction frequency in the study groups. RVDD – right ventricular diastolic dysfunction; *p < *0.05 compared with men*.

The right ventricle diastolic dysfunction was detected in the Shors as often as in the non-indigenous population (29.3% and 20.0%, *p* = 0.406). However, stratification by sex (Fig. 2) revealed significant differences (H = 11.81, *p* = 0.0081) primarily due to the more frequent detection RVDD in women compared with men (23.1% and 1.9%, *p* = 0.0014). At the same time, ethnic differences in RVDD between women and men wasn’t revealed.

In a univariate binary logistic regression including of all variables (Table 4), the following factors had a significant association with the right ventricle diastolic dysfunction (χ2(24) = 62.5, *p* < 0.001): ethnicity, sex, smoking, body mass index, obesity, systolic blood pressure, urea, LVMM index, diastolic LV function (e'/a', E/e'). The model explained 67.8% (Nagelkerke R2) of the variance in RVDD and correctly classified 90.1% of cases. In multiple binary logistic regression model (forward LR method), sex was the only significant factor included in the model (χ2 = 11.8, *p* < 0.001), but this model explained only 16.2% (Nagelkerke R2) of the variance in RVDD and correctly classified 74.3% of cases. Nevertheless, despite the reliable statistical significance of the latter model, its low quality (judging by the Nagelkerke R2 indicator) does not allow us to reject univariate analysis with other indicators associated with RVDD in , in particular, the ethnicity of the patients.

**Table 4:** Factors associated with the presence of right ventricular diastolic dysfunction (results from binary logistic regression analysis).

	B	S.E	Wald	df	Sig	Exp(B)
Caucasians ethnicity	− 3.479	1.148	9.179	1	0.002	0.031
Female sex	4.507	1.668	7.304	1	0.007	90.656
Age	− 0.007	0.068	0.012	1	0.913	0.993
Smoking	3.177	1.495	4.515	1	0.034	23.971
BMI	− 0.515	0.240	4.615	1	0.032	0.597
Obesity	4.110	1.883	4.763	1	0.029	60.951
SBP	0.163	0.067	5.863	1	0.015	1.177
DBP	− 0.012	0.091	0.019	1	0.891	0.988
Glucose	− 0.014	0.322	0.002	1	0.965	0.986
Cholesterol	− 0.371	0.591	0.395	1	0.530	0.690
Triglycerides	− 1.447	0.856	2.853	1	0.091	0.235
LDL	1.010	0.529	3.643	1	0.056	2.746
HDL	0.602	0.614	0.960	1	0.327	1.826
Urea	1.364	0.448	9.270	1	0.002	3.912
Creatinine	− 0.029	0.024	1.498	1	0.221	0.971
LVEF	− 0.193	0.111	3.021	1	0.082	0.824
TPWi	− 2.150	1.127	3.639	1	0.056	0.117
TIVSi	0.393	0.845	0.216	1	0.642	1.481
LVMMi	5.040	1.966	6.570	1	0.010	154.448
E/A	0.802	1.019	0.620	1	0.431	2.231
e’/a’	− 4.366	1.637	7.114	1	0.008	0.013
E/e’	0.809	0.289	7.830	1	0.005	2.245
Constant	− 4.282	10.258	0.174	1	0.676	0.014

SBP—systolic blood pressure; DBP—diastolic blood pressure, BMI—body mass index; LDL-low-density lipoprotein; HDL-high density lipoprotein; LVEF—left ventricular ejection fraction; TPWi—posterior wall thickness index, TIVSi—interventricular septum thickness index; LVMMi—LV myocardial mass index, E—early diastolic mitral flow (pulse Doppler); A—late diastolic mitral flow (pulse Doppler); e’ -, a’—early and late diastolic relaxation velocity of lateral mitral annulus (tissue Doppler).

**Figure 3:**
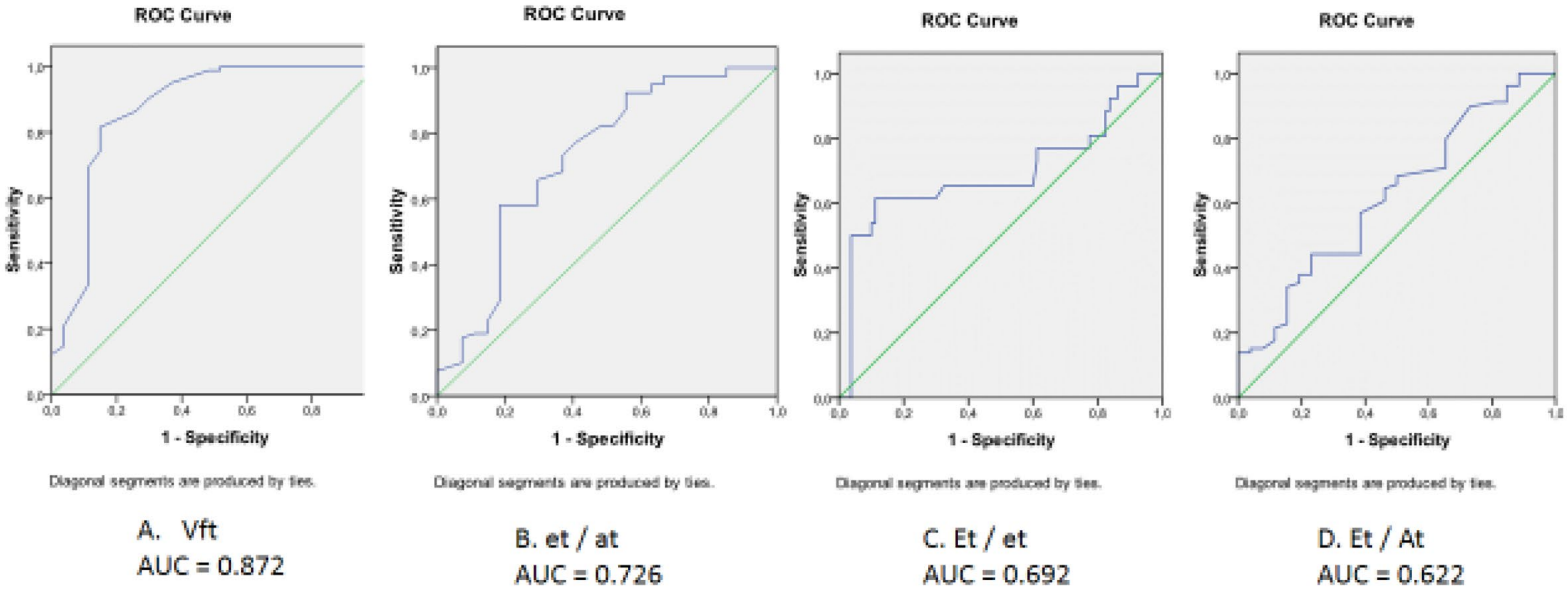
Receiver operating characteristic curve analysis. Performance efficacy of the right ventricle echocardiographic parameters in the right ventricular diastolic dysfunction detecting. *Vft—propagation velocities of the early tricuspidal flow, E*_*t*_*—early transtricuspid diastolic filling; A*_*t*_*—late transtricuspid mitral diastolic filling; e'*_*t*_*—early diastolic tricuspid annular tissue velocity; a'*_*t*_*—late diastolic tricuspid annular tissue velocity; AUC—area under curve*.

**Figure 4:**
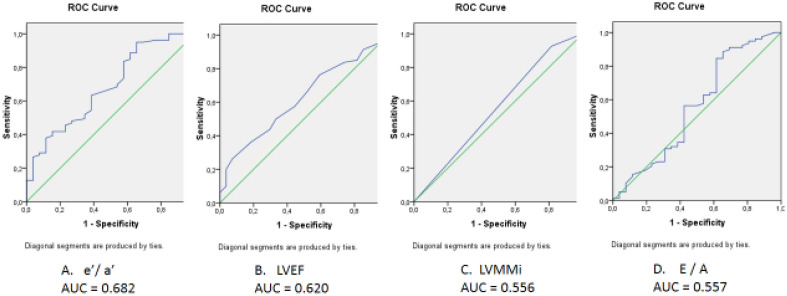
Receiver operating characteristic curve analysis. Performance efficacy of the left ventricle echocardiographic parameters in the right ventricular diastolic dysfunction detecting. *e’—early diastolic relaxation velocity; a’—late diastolic relaxation velocity; LVEF—left ventricular ejection fraction; LVMMi—LV myocardial mass index; E—early diastolic mitral flow (pulse Doppler); A—late diastolic mitral flow (pulse Doppler); AUC—area under curve*.

ROC-curves of the studied left ventricular variables (LVMMi, LVEF, ratio E/A and e’/a') association with RVDD are presented in Fig. 3. The areas under the curves were below 0.7 (for e’/a'—0.682, for LVEF—0.620, for E/A—0.557, and for LVMMi—0.556), which indicated an inability of these to identify RVDD. Among the right ventricle indicators (Fig. 4) the ability to identify RVDD was established for the velocity of early diastolic flow propagation (AUC = 0.872) and the ratio e't / a't (AUC = 0.726).

## Discussion

When assessing the right ventricle parameters in hypertensive patients we revealed lower values of the pulmonary artery index, the size and area of the right atrium, as well as the highest values of the RV velocity flow propagation, the rate of the tricuspid annulus early diastolic and systolic movement in Shor men compared with Caucasians. Shor women had the lowest values of early transtricuspid blood flow and the Et / At ratio. RV diastolic dysfunction was detected mainly in women, somewhat more often in Shor women. Accordingly, ethnicity was one of the factors associated with the RV diastolic dysfunction presence. Risk factors (smoking, obesity), blood pressure, gender, ethnicity, as well as LV parameters (impaired diastolic filling and an LV myocardial mass increase) were also associated with detection of RVDD.

Our previous study assessed the state of the right heart in healthy individuals of the Shor nationality^21^. In that study, an increase in the dimensions of the pulmonary artery and right ventricle was found in Shor men and women compared to Caucasians. The indicators of the RV diastolic filling in the Shors were moderately better, which was manifested, in particular, by higher RV filling velocity in them. Participants of the present study were older and, therefore, both arterial hypertension and age influenced the state of the right heart, leveling out the initial ethnic differences in the LV structural parameters observed when comparing healthy individuals, and the right heart indicators in Shor women. It is possible that genetic factors influenced this dynamic of indicators during hypertension development. It was previously shown that different ethnic groups of rural Gornaya Shoriya residents had different genetic associations with LV hypertrophy. At the same time, LV hypertrophy among hypertensive patients was more prevalent in the Shor group than in the non-indigenous (Caucasian) group^4^. In the previous study, no genetic associations with the state of the right ventricle were studied, however, in our study, there were no ethnic differences in the severity of LV hypertrophy. A possible reason for this is the influence of environmental factors: the shift from the traditional way of life of the Shors in rural areas to life in the city, greater availability of medical care, and an increase in the educational level. Examining healthy individuals in the MESA-Right Ventricle Study has shown that age, sex, and race are associated with significant differences in RV mass and volumes^19^. The authors suggested that these differences could potentially explain distinct RV responses to cardiopulmonary disease^19^; however, in our study, on the contrary, we noted the leveling of the initial ethnic differences in RV parameters during the development of arterial hypertension.

Previous studies have shown that the level of physical activity^22^, smoking^23^ and the left ventricular hypertrophy^12,24^ affect the right ventricle. It was previously shown that healthy residents of highlands have small left heart and large right ventricle due to exposure to hypoxemia at high altitudes, and these changes did not depend on ethnicity^25^. In the present study the RV dimensions are higher in Shor men than in non-indigenous men, which can be explained by a combination of several factors (high smoking frequency, genetic predisposition, and a decrease in daily activity due to changes in the traditional lifestyle is compensated, apparently, by the high prevalence of heavy physical labor among them).

We focused on the RV diastolic function since its development precedes systolic dysfunction both in experiment^26,27^ and during the disease’s development^10,28^, adversely affecting the prognosis at the same time^7,29^. Our study confirmed the influence of the above factors on the presence of RVDD. The detection rate of RVDD was significantly lower than in a number of previous studies, where it was up to 45–60%. However, these studies examined patients with stable coronary artery disease before surgery^10^ or with uncompensated hypertension^24^. The more frequent RVDD detection in women turned out to be unexpected for us. In previous studies, on the contrary, there was a greater resistance of women to the RV dysfunction development compared to men^30^. Perhaps this is characteristic of RV systolic dysfunction, but not of diastolic dysfunction. It also cannot be ruled out that the existing criteria for RV diastolic dysfunction may inaccurately reflect its presence in women, which apparently requires further research in this area.

Among the RVDD echocardiographic indicators, the velocity of RV filling to the greatest extent reflected its presence. This is probably a natural result. First, this indicator changes linearly with the increasing severity of RVDD, in contrast to the ratio of transtricuspid flow velocities. Secondly, assessing the filling flows of the right ventricle using 4D-MRI turned out to be the most informative in identifying initial changes in the right heart^31^. It is proposed to continue the study of the 4D-MRI technique in assessing the RV diastole^32^, but, apparently, it is impossible to leave out the echocardiographic assessment of the RV filling flows due to the greater availability of this examination technique. Furthermore, this is consistent with the notion that, due to its high availability, echocardiography is the first choice of imaging modalities for assessing the right ventricle. In turn, MRI should be performed as a second-line imaging modality in cases where surgery is planned for congenital heart disease or when differential diagnosis is needed^33^.

We see the clinical significance of the study in the fact that, firstly, the leveling of most ethnic echocardiographic differences between Shors and Caucasians in the development of arterial hypertension shows that the clinical assessment may not take the influence of the patient's ethnicity into account. Secondly, the obtained data emphasize the complex interactions of genetic factors, environmental conditions, development of diseases, as well as a change in the traditional lifestyle of the Shors (moving from the middle mountains to the plains, reducing daily physical activity, changing diet), increasing the availability and quality of medical care for them for changes in the right heart. Revealing the diastolic function of the right ventricle predominantly in women among hypertensive patients requires additional study.

Besides, the Shors are a small people, the total number is about 14 thousand people. However, they are close to other Turkic-speaking peoples living in Siberia (Altaians, Khakasses, Chulyms, etc.). Therefore, the results obtained by us can be useful for examining representatives of other small peoples of Siberia, as well as other representatives of the Mongoloid race in Asia.

### Study limitation

Several limitations should be mentioned. Subclinical coronary artery disease cannot be excluded in this study because coronary angiography was not performed. However, invasive diagnostics had not been indicated since this study included asymptomatic participants with no evidence of atherosclerotic lesions in other arterial regions. Another limitation is the relatively small number of included patients. This was due to the relatively small number of Shors living in urban settings. Nevertheless, we managed to obtain statistically significant results, which are desirable to confirm in larger studies. Finally, the assessment of right ventricular function was based on standard indicators of right ventricular systolic and diastolic function without the use of second-level methods (for example, right and left atrial atrioventricular strain), which have been used in recent years, including in patients with arterial hypertension^24,30^. However, an international study has shown that new technologies such as global longitudinal strain and 3D echocardiography are rarely used to quantify right ventricular function in clinical setting (3% and 1%, respectively)^34^. Therefore, the use of traditional RV indicators, in our opinion, at present can be justified, especially in an essentially screening study similar to ours. However, in the future, it is rational to conduct research using new technologies for assessing ethnic differences in RV function.

## Conclusion

Our study established the influence of ethnic differences on the right heart echocardiographic parameters in Shors and Caucasians with arterial hypertension. We revealed in Shor men the lowest values of the pulmonary artery index, the size and area of the right atrium, as well as the highest values of the RV velocity flow propagation, the rate of the tricuspid annulus early diastolic and systolic movement in comparison with Caucasians. Shor women had the lowest values of early transtricuspid blood flow and the Et / At ratio. RV diastolic dysfunction was detected mainly in women, somewhat more often in Shor women. Accordingly, ethnicity (Shors) was one of the factors associated with the RV diastolic dysfunction presence. The revealed differences should improve the assessment of the structure and function of the right heart in patients with arterial hypertension of minor ethnic groups, which help to improve the diagnosis and management of such patients.

## Data Availability

The datasets used and/or analyzed during the current study available from the corresponding author on reasonable request.
